# Omic personality: implications of stable transcript and methylation profiles for personalized medicine

**DOI:** 10.1186/s13073-015-0209-4

**Published:** 2015-08-13

**Authors:** Rubina Tabassum, Ambily Sivadas, Vartika Agrawal, Haozheng Tian, Dalia Arafat, Greg Gibson

**Affiliations:** Center for Integrative Genomics, School of Biology, Georgia Institute of Technology, Boggs Building 1-96, 770 State Street, Atlanta, GA 30332 USA; Institute for Molecular Medicine Finland, Helsinki, Finland; CSIR-Institute of Genomics and Integrative Biology, Delhi, India; Philips Health Care, Briarcliff Manor, NY USA

## Abstract

**Background:**

Personalized medicine is predicated on the notion that individual biochemical and genomic profiles are relatively constant in times of good health and to some extent predictive of disease or therapeutic response. We report a pilot study quantifying gene expression and methylation profile consistency over time, addressing the reasons for individual uniqueness, and its relation to *N* = 1 phenotypes.

**Methods:**

Whole blood samples from four African American women, four Caucasian women, and four Caucasian men drawn from the Atlanta Center for Health Discovery and Well Being study at three successive 6-month intervals were profiled by RNA-Seq, miRNA-Seq, and Illumina Methylation 450 K arrays. Standard regression approaches were used to evaluate the proportion of variance for each type of omic measure among individuals, and to quantify correlations among measures and with clinical attributes related to wellness.

**Results:**

Longitudinal omic profiles were in general highly consistent over time, with an average of 67 % variance in transcript abundance, 42 % in CpG methylation level (but 88 % for the most differentiated CpG per gene), and 50 % in miRNA abundance among individuals, which are all comparable to 74 % variance among individuals for 74 clinical traits. One third of the variance could be attributed to differential blood cell type abundance, which was also fairly stable over time, and a lesser amount to expression quantitative trait loci (eQTL) effects. Seven conserved axes of covariance that capture diverse aspects of immune function explained over half of the variance. These axes also explained a considerable proportion of individually extreme transcript abundance, namely approximately 100 genes that were significantly up-regulated or down-regulated in each person and were in some cases enriched for relevant gene activities that plausibly associate with clinical attributes. A similar fraction of genes had individually divergent methylation levels, but these did not overlap with the transcripts, and fewer than 20 % of genes had significantly correlated methylation and gene expression.

**Conclusions:**

People express an “omic personality” consisting of peripheral blood transcriptional and epigenetic profiles that are constant over the course of a year and reflect various types of immune activity. Baseline genomic profiles can provide a window into the molecular basis of traits that might be useful for explaining medical conditions or guiding personalized health decisions.

**Electronic supplementary material:**

The online version of this article (doi:10.1186/s13073-015-0209-4) contains supplementary material, which is available to authorized users.

## Background

This study is an enquiry into the stability of omic “personalities”, namely, the degree to which functional genomic profiles such as the transcriptome and methylome retain individual-specific features over time. Just as humans are able to recognize one another through facial and other morphological (and behavioral) attributes that remain person-specific for decades, we ask whether omic profiles have a similar capacity to define personalities at the molecular level. If functional genomic data types are to be incorporated alongside genome sequences as a component of clinical medicine [1, 2], it is essential that we have a baseline understanding of whether diagnostic and transcriptional, protein, metabolite, and epigenetic biomarkers remain stable over time [3–5], whether perturbation in the context of disease is transient or permanent [6], or if behavioral interventions can push profiles to a more healthy state.

The dataset we describe allowed us to assess the stability of omic profiles in healthy volunteers who were part of a wellness intervention study at Georgia Tech and Emory Universities, the Center for Health Discovery and Well Being (CHDWB) [7, 8]. It consists of RNA sequencing (RNA-Seq), micro-RNA sequencing (miRNA-Seq), and DNA methylation (Illumina Methylation 450 K array) data from whole blood sampled three times at 6-month intervals from each of 12 participants. Whole genome sequences and clinical values for 74 traits, also measured at the three time points, were also available and have been previously described for the eight European Americans (four men and four women) in the study [9]. We added data for four African American women. All individuals were chosen to represent a diversity of physical and mental health phenotypes. For the most part, the clinical attributes did not change appreciably over the course of the study [10] and three quarters of the variance in each trait tended to be among individuals.

Two “straw man” alternative *a priori* models for the extent of omic personality may be postulated. The first is that gene expression and methylation are sufficiently labile that multiple measures from any individual are unlikely to be identifiable as belonging to one person. This may be because blood cell type abundance fluctuates, environmental exposures change, and people experience different health states from month to month and week to week. For example, considerable lability of the methylome has been observed in the first two years of life [11, 12], as well as with advancing age [13–18]. The opposite model would be that our omic profiles are just as stable as our visible phenotypes, and that clustering of any individual samples for any of the three data types would lead to side-by-side alignment for the three samples for each person. Our analyses unambiguously favor this latter model, at least over a 12-month period, consistent with the notion that people have strong omic personalities. Within this model, three further sub-models may be considered. One is that the individual-biased expression is restricted to a limited number of genes that have relatively strong deviations, perhaps due to cis-acting regulatory effects that are known to explain up to 30 % (and in some cases more) of the variance of individual transcripts among people [19–22]. Alternatively, it could be distributed over the majority of transcripts. The third possibility is that the individual-biased expression is highly structured such that covariance of hundreds or thousands of transcripts along a limited number of axes of variation explains much of the individual specificity, rather than each gene being independently regulated [23, 24]. This would imply that trans-acting factors are more important than cis-regulatory ones in defining a person’s omic personality.

A number of early microarray studies explored the individuality of gene expression and its relationship to blood cell counts. In 2003, Whitney et al. [3] noted suites of genes associated with lymphocyte, neutrophil, and reticulocyte abundance (which essentially correspond to Axes 1, 5, and 2 in our study defining conserved axes of covariance in blood [21]), but only documented 340 genes with high “intrinsic scores” in peripheral blood monocytes, implying that they were differentially expressed among 16 individuals. By contrast, Eady et al. [5] took a more standard statistical approach and argued for individualized expression of over 3,300 genes (39 % of those represented on their microarrays) in a study of 18 adult volunteers sampled weekly over a month. Studies of methylation in peripheral blood have documented much stronger correlations than transcripts with age [13, 14], as well as with gender and body mass index at many loci, but it appears that the modular structure of methylation is generally not correlated with that of the transcriptome [13, 25]. Here, we quantify the correspondence between gene expression and DNA methylation profiles in 12 adults over a year, also relating the observations to clinical attributes of the study participants. The data lead us to argue that steady-state omic profiles may well prove to be useful in personalized medicine as markers of individual health status.

## Methods

### Ethics, consent, permissions and consent to publish

We studied the profiles of 12 middle-aged individuals (39–61 years old) chosen to represent a range of clinical profiles in the CHDWB study [7, 8], including four African American women (Aa, Ab, Ac, Ad), four Caucasian women (Ce, Cf, Cg, Ch), and four Caucasian men (Mi, Mj, Mk, Ml). The individuals all consented under the institutional review boards approvals of both Emory University and Georgia Tech to analysis of their gene sequences, transcriptome, and epigenome, including the permission to publish such data. The research adheres to the tenets of the Helsinki Declaration and is consistent with all relevant local regulations in Atlanta, GA, USA. However, the individuals do not currently consent to the right to receive feedback of their own genomic data, and hence their identities are protected. The same Caucasian individuals were reported in our previous study of genotypic and clinical risk of disease [9], but with different identifiers, again to protect privacy. All data are available after approval by request to the Data Access Committee of the CHDWB, while the gene expression and methylation profiles are available at the Gene Expression Omnibus [GEO:GSE67491].

### Sample collection

Peripheral blood samples (10 ml) were collected into EDTA tubes that were frozen for DNA preservation, and Tempus RNA tubes (Life Technologies, Grand Island, NY, USA) for preservation of RNA. Samples were taken during regular 6-monthly visits to the Center, generally between the hours of 10:00 a.m. and 12:00 noon. The 74 clinical traits considered here are listed in Additional file 1: Table S1 and were measured within 2 h of the blood sampling or generated from self-reported survey assessments taken within 1 week of each sample. A total of 668 individuals enrolled and have participated in the CHDWB, which is an ongoing longitudinal study designed to evaluate the impact of health coaches on wellbeing. Across the cohort, significant improvement in most major indicators of health including physical, biochemical, and mental health parameters is observed [10], and is maintained for at least 3 years, but the effects are modest and tend to be restricted to those individuals with the highest baseline risks.

### RNA-Seq

Whole blood transcriptomes were characterized by RNA-Seq using paired-end 100 base pair (bp) sequencing on an Illumina HiSeq2000 (Illumina Inc, San Diego, CA, USA) at the Vanderbilt University Medical Center in 2012, with four samples per lane, randomized with respect to individual and time point. Unstranded TruSeq cDNA libraries were prepared using Illumina TruSeq RNA Sample Preparation Kits (Set A, v2) from total RNA isolated from Tempus tubes following the manufacturer’s protocols. For eight of the individuals, we replicated the sequencing of the same libraries using the Yerkes NHP Genomics Core Laboratory (Atlanta, GA, USA) in 2013. In all cases, each individual’s replicated profile clustered side-by-side with the initial sample, so the reads were combined to produce an average coverage of approximately 50 million paired-end reads (range 40 million to 80 million), with average read depth of 40× at the exons. As an additional quality check, we also contrasted the RNA-Seq profiles with microarray results available for the baseline samples, observing complete concordance in the identity of samples with the highest correlations across platforms.

All short reads were aligned to HuRef19 using the TopHat spliced-reads fast mapper in Cufflinks v2.0.1 [26]. Downstream analyses were performed at the level of the whole gene, using the Supervised Normalization of Microarray (SNM) algorithm [27] to normalize the log2 transformed read counts (fragments per kilobase of transcript per million mapped reads, FPKM) with a model fitting individual as the biological variable, and removing effects of RNA quality and ethnicity. A total of 11,265 expressed genes were analyzed, using FPKM > 1 as the threshold for detection in all samples.

### miRNA-Seq

miRNA-Seq was similarly performed using small RNA prepared from the identical whole RNA samples, with Illumina TruSeq Small RNA Sample Preparation Kits, running 18 samples per lane for an average of 7 million reads. Six samples failed, one each for individuals Aa, Ad, Ch, and Mk, and two for Ac. Analysis of sequence reads was performed using miRExpress [28, 29]. The log2 transformed reads per million counts were normalized by the SNM algorithm using the same model as for RNA-Seq data and were used for downstream analyses.

### Methylation

Whole blood DNA obtained at the same time as the RNA samples was subject to methylation profiling using Illumina Human Methylation 450 K BeadChip arrays following bisulfite conversion. The samples were processed by the Illumina Genome Network laboratory at the University of Washington in parallel to the generation of whole genome sequence data, with all samples processed concurrently to avoid batch effects [30]. These arrays provided comprehensive coverage of over 450,000 CpG sites distributed intergenically, in CpG islands and shores, and in gene bodies, and within 1.5 kilobases (kb) or 200 bp of the annotated transcription start site (TSS) of most human genes. Other annotations include whether the CpG sites occur within DNase hypersensitive sites, or are known to be differentially methylated in tumor versus normal or across a variety of tissue types. Methylation levels were expressed as beta values ranging from 0 (non-methylated) to 1 (fully methylated) obtained directly from the GenomeStudio output (Illumina, Inc.) from scanning the bead arrays. Further transformations including mean centering and quantile normalization were explored, but all data reported in this manuscript refer to the raw beta values deposited at GEO. Regrettably, each of the three samples for each individual were processed on the same chip, so there is confounding of individual with chip, as documented in the experimental design file Additional file 1: Table S2. However, chip explained less than 10 % of the variance, one fifth of that explained by individual, and permutations imply that this is expected by chance. Because principal component 1 (PC1), which differentiated individuals, was not associated with slide, we elected not to adjust the chip effect to avoid overfitting. Only autosomal probes were considered for most analyses. Islands refer to CpG sites situated within 200 bp or greater stretches of DNA with more than 50 % CG content and an excess of CpG; shores refer to CpG sites within 2 kb on either side of islands, and shelves refer to an additional 2 kb further from an island than shores.

### Allele-specific expression

Allele-specific expression was estimated by generating the counts for each short read aligned to either the HuRef19 human reference genome or to a proxy genome sequence (referred as alternate genome) generated for each individual containing the alternate allele at each heterozygous site from their own whole genome sequence [9]. Allele-specific expression was estimated only for sites that were confirmed heterozygous in the individual’s whole genome sequence, and for which at least eight reads were available containing the polymorphic site in all three biological replicates. We chose eight reads to compromise between including sufficient sites for the comparison (greater depth would exclude sites and individuals) while minimizing the impact of underestimation and overestimation of biases (three, four or five out of eight produce ratios of 0.375, 0.5, and 0.625 but are within sampling variance, whereas three measures of four out of eight would underestimate variance). Notably no correlation between read depth and bias was observed at this level of inclusion. Three individuals (Ac, Ad, and Ml) had fewer than 13,000 comparable sites after applying these filters and were excluded from the analysis, which was thus restricted to nine individuals. Preliminary analysis shown in Additional file 2: Figure S1A for individual Aa indicated strong tendency for under-representation of the alternate allele in alignment to the reference genome, whereas under the null the average representation of each allele is expected to be 50 % (assuming that transcript abundance is independent of allele frequency and hence representation in the reference genome); the average in most individuals was 47.4 %. This reflects in part the inclusion of probes that are known to map differentially as reported by others [22]. Rather than excluding such probes, we noted that there was a continuum of differential alignment biases, so we took an empirical approach. Comparison of the alignments to the alternate and reference genomes indicated that two thirds of the heterozygous sites had an average 5 % or more difference in alternate allele frequency between alignments, and only 10 % of sites had an estimated alternate allele frequency that differed by less than 2.5 %. Utilizing the alternate genome alignments results in a slight excess of expression biased toward the alternate allele (average 51.6 %), but with an approximately symmetrical distribution of biases. Additional file 2: Figure S1B contrasts the estimated biases for the reference and alternate genomes for Aa, highlighting the strong underestimation of bias for around 5 % of probes.

Although we report results across the full spectrum of alleles, the proportion of sites that were significantly biased in both the alternate and reference alignments was almost the same regardless of the absolute concordance between the two alignments. Single nucleotide polymorphisms (SNP) rs9906320 in Aa illustrates the problem: the proportions of the alternate allele in the three replicates when aligned to the reference genome were 0.39, 0.43, and 0.44, but when aligned to the alternate genome were 0.51, 0.51, and 0.51, so an allele-specific expression (ASE) bias would be inferred in the reference alignment but not in the personalized alignment. Transcriptome-wide, this typically translated to a 4-fold excess of incidence of reference allele bias in alignments to the reference genome, and a 2.5-fold excess of alternate allele biases in alignments to the alternate genome, though both yielded estimates of 10 % of sites showing ASE at the 5 % significance level. Restricting the analysis to sites that had similar estimates in the two alignments did not meaningfully change the proportions, so had no qualitative impact on the conclusions regarding the individual-specific level of ASE and their repeatability across individuals.

### Statistical analyses

**Fig. 1:**
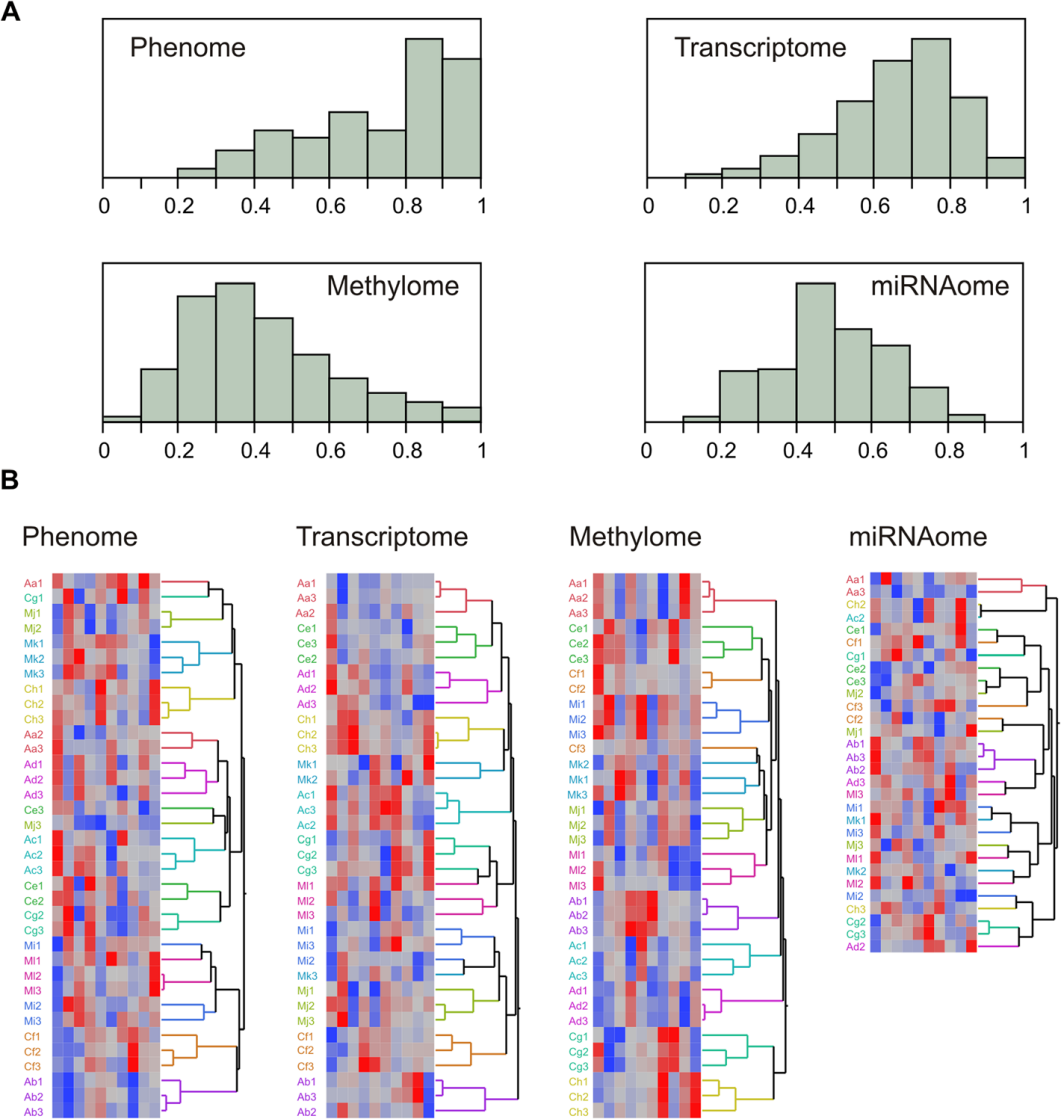
Partitioning of omic profiles among individuals. **a** Each histogram shows the density of the percent variation for each measure (feature) in the indicated omic class that is among individuals, based on three biological samples taken at 6 month intervals for 12 people. Pearson correlation R-squared values were obtained from standardized trait measures (phenome, transcriptome or miRNAome), or the beta values (methylome, since these are not normally distributed). **b** Two way hierarchical clustering of the first 10 principal components of the 36 samples, showing strong conservation of the summary measures within individuals across replicates, with the exception of the miRNAome. The ten columns in each panel correspond to the first 10 PC, blue negative, red positive values

Statistical analyses were performed in SAS/JMP Version 5 (SAS Institute, Cary, NC, USA) or R, and simple correlations were assessed in Microsoft Excel. Principal component analyses reported in Fig. 1 and Additional file 1: Table S3 were performed by computing the first ten PCs for each of the four omic data types (clinical, mRNA, methylation, and miRNA) across all 36 samples (30 for miRNA). The scree plots of the amount of variance explained by each PC differed for each data type, suggesting that different numbers of PC were significant. Because alternative statistical methods yield slightly different cutoffs, and clustering algorithms also vary in their grouping of samples, we chose to report the first ten PCs as these generally capture 80 % of the variance, and to use Ward’s method for the hierarchical clustering However, note that for the clinical data, perfect clustering of all 12 individuals was seen with eight PCs [73.2 % variance explained (VE)], while the addition of PC10 added noise, causing the slight reordering seen in the Phenome panel of Fig. 1b. For the transcript data, maximal individual clustering was attained with ten PCs as shown, but eight individuals grouped uniquely with as few as five PCs, as well as with eight PCs (75.6 % VE). For the methylation data, stable clustering of ten individuals was seen with eight PCs (67.7 % VE), and this did not improve or change as more PCs were added. However, for the miRNA data, no more than two individuals clustered together no matter how many PCs were included. With five PCs, the numbers of individuals clustering uniquely was eight for clinical data, eight for mRNA data, eight for methylation data, and one for miRNA data, with 57.8, 63.1, 50.2, and 59.0 % VE, respectively.

R-squared values for all estimates of the amount of variance among individuals were estimated as the square of the Pearson correlation coefficient reported by SAS/JMP. Technically, because each individual was measured at three different times, the within-individual measures were not true biological replicates, so are referred to as pseudo-biological replicates. Because there were no degrees of freedom to assess the time-within-individual component of variance, we chose not to report the intra-individual variance because high values may simply have been due to single sample measurement or technical error. However, readers should note that the technical noise contribution to the variance within and among individuals has not been estimated, and the among-individual effects from the R-squared values are likely an overestimate of the true differences between individuals.

Differential expression or methylation was computed by analysis of variance (ANOVA), namely from the F-ratio of the variation within individuals over the three visits, to the total variance in the 36 samples. We controlled for multiple comparisons by setting the *p*-value at 10^−4^ for identifying extreme features in each individual, resulting in a small false discovery rate (<5 % for all except individual Cg. A common false discovery rate (FDR) criterion for all individuals would have resulted in the inclusion of more genes in both analyses, so our analysis can be seen as conservative, but was favored as more consistent given that all analyses used 11 of the same 12 individuals for the comparisons.

## Results

### Longitudinal conservation of omic profiles

Individuals differ with respect to their phenome, transcriptome, epigenome, and miRNAome, both at the level of single measures and whole profiles. In order to quantify these differences, we first calculated the percentage of the variance in traits, transcripts, CpG methylation levels, and miRNA abundance among individuals, and then performed hierarchical clustering on the PCs of variation to visualize profile conservation. Both sets of observations, summarized in Fig. 1, imply that omic individualization in human blood is approximately as high as phenotypic differentiation among people, namely, that differences at the molecular level are generally highly stable over a 12-month period. Panel b of Fig. 1 reports results of hierarchical clustering of individuals with the first ten PCs, which maximized the concordance of individuals for the transcriptome; but, as explained in “Methods,” the use of eight PCs maximized the concordance of individuals for clinical traits.

The amount of variance for 74 standardized clinical measures among individuals ranged from 25 % (Beck Depression Index) to close to 99 % (body mass index), with a mean of 74 % and standard deviation of 19 %. Half of the traits had over 80 % of the variance among individuals, and, correspondingly, each individual’s overall trait profile remained quite constant over the three visits spaced at 6-month intervals. For seven of the 12 individuals, hierarchical clustering of the first ten PCs resulted in the three profiles clustering together (Fig. 1b, Phenome). For the remaining five individuals, either just the baseline or third visit was slightly divergent. If the clustering was performed on the actual trait values (not shown), the similarity was even stronger, and all 12 individuals clustered uniquely, reflecting the constancy of the metabolic traits in particular.

Similarly, for the transcriptome, the mean amount of variance among individuals was 67 % with a standard deviation of 15 %. One fifth of the transcripts (2,212/11,265) had more than 80 % of the variance among individuals. This implies that inter-individual variation is not restricted to a small number of genes but rather is observed across the full spectrum of transcript abundance. Because there was also considerable covariance of transcript abundance, the first ten PCs captured 81.5 % of the variance, and hierarchical clustering on these PCs resulted in adjacency of all three time points for 11 of the 12 individuals (Fig. 1b, Transcriptome). The same result was observed upon clustering of the individual transcripts, providing a strong demonstration of the constancy of transcriptomic profiles in essentially healthy individuals.

The methylome also told a similar story, although the conservation was not quite as strong. Figure 1a shows that considering all 356,119 autosomal CpG sites indicated by Illumina annotation to lie in the vicinity of genes, the average amount of variance that was among individuals was just 42 % (standard deviation 21 %), yet one third of the sites (127,980) had more than half their variance among individuals. A similar analysis of just the most differentiated site within 9,468 expressed genes indicated a mean of 76 % variance among individuals (not shown), implying that at least some CpG sites within almost all active genes have divergent methylation levels that are stably maintained. Clustering by individual according to the first ten PCs of all of the CpG sites, explaining 80 % of their variance, again led to adjacency of all three time points for 11 of the individuals (Fig. 1b, Methylome). Only slightly less concordance was seen when clustering on the individual CpG methylation levels.

Analysis of the miRNA abundance was compromised by poor quality libraries for six of the samples. Nevertheless, there was a clear reduction in the level of among-individual variance relative to the transcriptome, with a mean of 50 % and standard deviation of 15 %. Correspondingly, there was considerably less consistency of the profiles, with the profiles for just one of the seven individuals for whom data was available at all three time points clustering together. Five others had at least two adjacent samples, suggesting that profiles are to some extent individual-specific but can fluctuate over time. The reduced individuality of the miRNA profiles may be an artifact of the data quality, but down-sampling of the RNA-Seq data to similar sampling levels as that observed for the miRNA retained the individuality documented above for the full transcriptome. In addition, the amount of variance among individuals for transcript abundance was constant across the full range of expression levels, with the exception of an average 5 % increase for the most highly expressed decile of transcripts, confirming that low read depth alone was not responsible for reduced inter-individual variance. We conclude that miRNA abundance is less tightly regulated than the mRNA.

### Causes of inter-individual variation in transcript abundance

There are multiple potential reasons for the high inter-individual variability in gene expression, including cis-eQTL effects, epigenetic modulation, and trans-regulation. We considered each of these in turn.

**Fig. 2:**
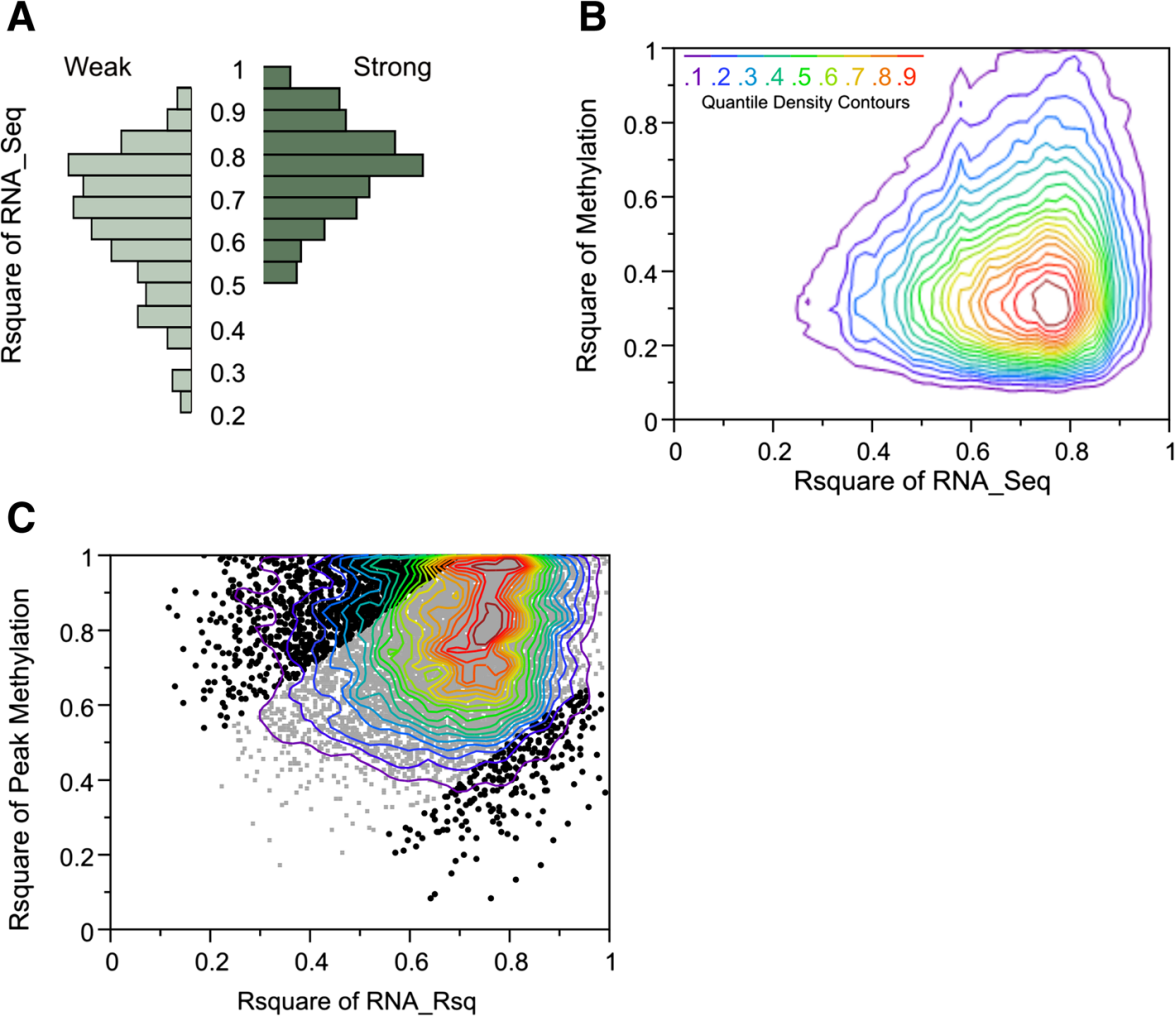
Genetic and epigenetic influences on inter-individual transcriptional differentiation. **a** The two histograms show the percent variation among individuals for the 1,000 transcripts that had the highest documented cis-eQTL effects (*Strong*) and 1,000 with only weak cis-eQTL (*Weak*). The highly significant shift in the distribution to higher R-squared values in the strong set is consistent with cis-eQTL contributing at least a portion of the inter-individual variability. **b** Density contours of the regression of the among-individual R-squared for transcript abundance on the among-individual R-squared for all CpG annotated to the vicinity of the same gene (within 1.5 kb of the transcription start site or in an exon). The methylation R-squared for the vast majority of CpG was less than the transcript R-squared. **c** Density contours and individual gene points for the same regression as in (**b**), but only with the peak CpG per gene, namely the site with the largest variance among individuals. *Black points* have residuals of at least 0.3 beta units

eQTL are defined as SNPs that associate with the abundance of transcript and are usually defined by linear regression of expression level on genotype in a large sample [31]. We focused here on cis-eQTL (namely, those which act locally on a transcript within 1 Mb on the chromosome). With just 12 individuals, most genes have fewer than two minor allele homozygotes even at common SNPs, so direct estimates from regression would be unreliable. Instead, we simply asked whether genes with already known eQTL effects in blood were more likely to vary among individuals. We extracted the top 1,000 genes from the blood eQTL browser [21] and compared the percent variance explained by individual differences with that for the 1,000 genes with the smallest eQTL effects, truncated at *p* < 0.001 (at which there is an approximate 25 % false discovery rate) and corresponding to the 6,700^th^ to 7,700^th^ ranked eQTL genes. Figure 2a shows that there was a highly significant difference between the two groups, with a mean variance explained by individual of 74 % for the strong-eQTL group, and 65 % for the weak-eQTL group (ANOVA, *p* < 10^−30^). The latter percentage is the same as the mean among-individual variance for all transcripts, implying no evidence for locally acting common regulatory variants accounting for the differential expression of those genes. There was also a tendency for more strongly expressed genes to have higher inter-individual variability (average R-squared for the top quintile of mean abundance was 0.72, compared with 0.65 for the other quintiles), which may have contributed to the variance explained by the strong eQTL. However, these results are fully consistent with the expectation that cis-QTL do explain in the range of 10–30 % of the variance among individuals for up to a quarter of transcripts [32].

The impact of methylation on individualized gene expression was assessed in two ways, first by contrasting the amount of variance explained by CpG status, and more directly by examining the correlation between methylation and transcript abundance for each gene. First, we noted that R-squared values for the individual effect of methylation level were quite similar for different classes of CpG (Additional file 1: Table S4), ranging from a mean of 0.43 for those in a shelf to 0.48 for those in a shore, and 0.46 for those in islands. Sites in the 3′ untranslated region (UTR) were slightly less likely to explain individual variability (mean R-squared = 0.44) than those in gene bodies (0.46) or within 1.5 kb of the TSS (0.47), while, surprisingly, intergenic CpG had the highest inter-individual variability (0.52), all with similar standard deviations just greater than 0.2. Known differentially methylated regions among tissues (0.55) were significantly more likely to be differentially methylated than all other sites, but CpG in enhancers did not show such a tendency overall. These analyses excluded the sex chromosomes.

Regression of the amount of variance among individuals for methylation on that for expression of the same gene was significant, but extremely weak overall (Fig. 2b). Of 184,294 methylation R-squared values, 13.4 % were greater than the corresponding transcript abundance R-squared values, which was 2 % less than that observed in permutations. Consequently, there was a slight tendency for genes with elevated methylation among individuals to also be differentially expressed, but the vast majority of differential expression did not correspond with altered methylation. Considering just the CpG with the greatest individual differentiation within each gene (which we refer to as peak CpG, Fig. 2c), two thirds of these had an inter-individual methylation R-squared value that was greater than the corresponding transcript (6,333 of 9,468). These were also more likely to be in promoter-proximal regulatory regions; whereas 18 % of all measured genic CpG were between 1,500 and 200 bp of the TSS (while another 15 % were within 200 bp of the TSS), 28 % of the peak CpG were annotated to this TSS1500 group, the increase coming mostly at the expense of the gene body CpGs whose proportion dropped from 43 % to 34 %.

**Fig. 3:**
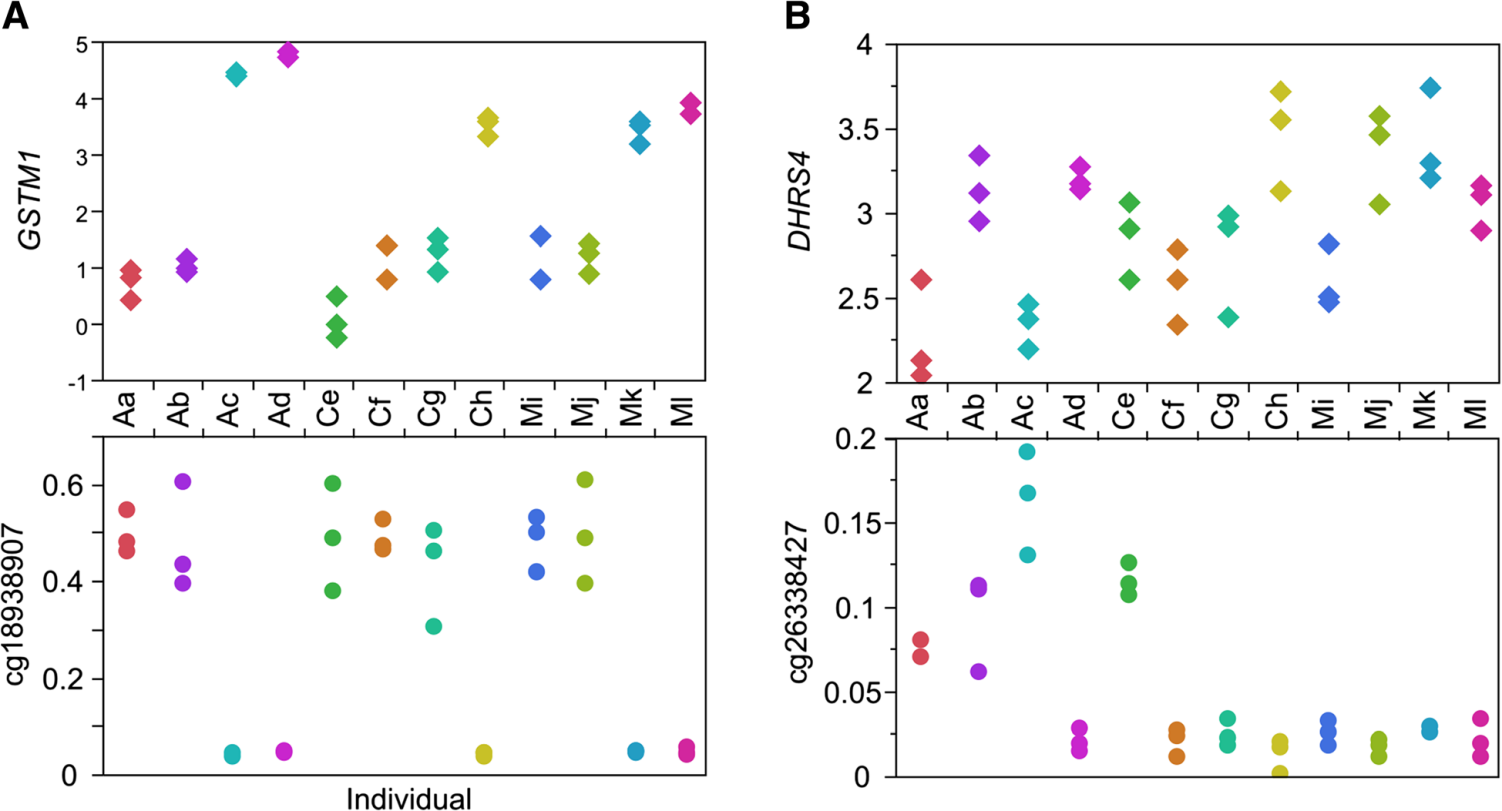
Examples of the relationship between methylation and gene expression where both measures are highly differentiated among individuals. **a** cg18938907 in *GSTM1* shows a strong correlation between high expression and hypomethylation . **b** cg26338427 in *DHRS4* shows partial correlation between low expression and hypermethylation

In order to assess whether peak CpG explain the transcript abundance, we computed the correlation between methylation level and transcript abundance. Across all 184,294 autosomal genic sites, just 1.6 % showed a significant relationship with an absolute value of the correlation coefficient greater than 0.5. However, 17 % of the 9,468 peak CpG were in either tail, implying that methylation status for at least one site for nearly one fifth of genes is strongly associated with gene expression. Additional file 1: Table S5 lists the strongest correlation for 1,710 genes for which there was good evidence that at least one methylation site associates with longitudinally consistent gene expression. A prominent example is shown in Fig. 3a, *GSTM1*, methylation of which has been proposed as a biomarker for predicting response to some types of cancer therapy [33]. The CpG island site cg18938907 is located in the first exon of *GSTM1*, and in each of five individuals with elevated expression was unambiguously hypomethylated.

It is further noteworthy that in approximately one quarter of cases where there was a strong correlation between inter-individual methylation and gene expression, at least one other site in the same gene was also correlated with transcript abundance. In one third of these cases, one CpG was positively correlated and another negatively correlated. In all cases, other CpG remained uncorrelated. Figure 3b shows a more typical situation for *DHRS4* where both the peak methylation site and the transcript were highly differentiated among individuals, but the two measures were not strongly correlated. In this example, two of the four individuals with elevated methylation at cg26338647, a promoter-proximal CpG island site, had very low gene expression whereas the other two individuals had normal expression. In the great majority of cases, though, there was no relationship at all between strongly differentiated methylation and differential gene expression [34]. We conclude that, as with the cis-eQTL, there is a minority of up to one fifth of genes where methylation of a subset of the CpG explains a substantial proportion of the variable transcript abundance. Though the effects in these cases are stronger than the genotype effects, differential methylation usually does not predict differential expression, and in no case should the strong association of methylation with gene expression be interpreted as simple causation.

Three potential trans-acting sources of variation are differential counts of major blood cell types, trans-eQTL, and cumulative regulatory effects that generate seven common axes of covariance of transcript abundance in blood [24]. Because there was no power to detect trans-eQTL in this sample, and the major loci were not expected to explain more than a few percent of the variance [21], each only influencing a small number of transcripts, they were not considered further.

Blood cell counts are highly differentiated among individuals, but explain only a modest proportion of the transcriptional variance [3–5]. In this study, over 80 % of the variance was among individuals for lymphocyte, monocyte, neutrophil, red blood cell, and platelet counts per milliliter of blood, but these five measures collectively accounted for an average of just 35 % of the variance of individual transcripts (range 1–90 %, standard deviation 16 %) when fit to a multivariate regression (Additional file 1: Table S6).

Much more of the transcriptional variance was explained by seven previously identified axes of variation, each of which was defined as the first PC of ten “blood informative transcripts” [24] that were the most tightly co-regulated of hundreds of correlated transcripts. More than two thirds of the variance was among individuals for six of the seven axes (the exception being Axis 4, 57 %) with a maximum of 94 % for Axis 7, which was enriched for genes involved in interferon responses. Note that Axes 1 and 5 were highly correlated with lymphocyte and neutrophil counts, but the other axes did not correspond to white blood cell counts. Collectively, the axes explained an average of 63 % of the variance in transcript abundance, similar to the amount they explain in large cross-sectional population studies, and almost twice as much as cell counts alone. These correlations are presented in Additional file 1: Table S7.

### Individual-specific loss and gain of transcript abundance and methylation states

**Fig. 4:**
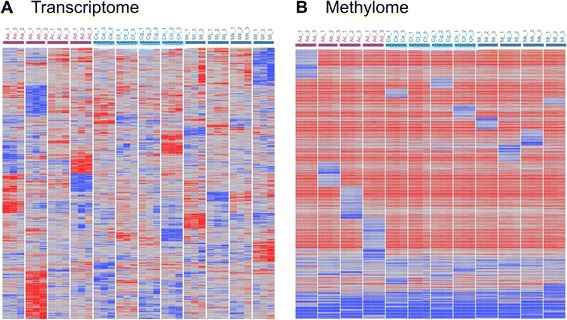
Extreme deviations in gene expression or methylation. **a** Heat map of all genes significantly differentially expressed in each individual relative to the other 11 individuals by ANOVA. Each of the three biological replicates for each individual clustered adjacent to one another, and groups of transcripts with elevated expression (*red*) or reduced expression (*blue*) are clearly visible. In some cases, there were reciprocal patterns for the most extreme sets, for example Aa and Ad or Mk and Ml. **b** Heat map of all peak CpG where one individual was at least 0.3 beta units deviant from the sample average. Each individual had a unique set of hypomethylated sites (*blue*, with an excess in the African American females Aa, Ab, Ac, and Ad), with relatively few hypermethylated sites, and a large number of sites where the distribution of beta values was bimodal with multiple *red* or *blue* individuals

The regulatory equivalent of deleterious coding variants is extreme loss or gain of transcript abundance. Because it is well known that each individual carries a burden of rare coding mutations, we sought to establish whether there is a similar gene expression burden by performing gene-specific one-way ANOVA contrasting each individual’s three pseudo-biological replicates against those of the other 11 individuals. With approximately 10,000 expressed genes, just one would be expected per individual at *p* < 10^−4^, so we took this as our significance threshold allowing for a small false discovery rate. A total of 1,227 genes were observed to be expressed in an individual-specific manner by these criteria, 694 lost and 533 gained (these are listed in Additional file 1: Table S8). Figure 4a is a heat map illustrating this individual-biased expression. As indicated in Table 1, one individual (Caucasian female Cg) had just a single aberrant transcript, while two (African American females Aa and Ad) had more than twice the average losses.

**Table 1:** Clinical associations with extreme expression

Person	Loss	Gain	Total	Notable loss	Notable gain	With Axis	Plausible association
Aa	289	55	344	*GATA2*, *ELK1*, *GSTM4*, *GPX4*	*KCNJ2*, *PTGR2*, *F8, LMNB1*	Low Axis 1	Calcitrol responsive genes with low blood calcium
							Low Axis 1 with low lymphocyte count
Ab	40	38	78	*SLC2A9*, *ITGB2*	*TUBB1*, *CD59*	High Axis 7	Diabetic: low sugar transporters with high blood glucose
							Predicted homocystinurea risk with low glutathione and cysteine-glutathione
							High tubulin 1 with high platelet count
Ac	38	17	55	*ALS2*, *SMPD1*			
Ad	128	61	189	*FUCA1*, *FUT4*, *FUT7*, *CA2*	*CR2*, *CD19*, *CD72*, *RETN*	High Axis 3	Low *CA2* with sleep disorder
							High Axis 3 with B cell activation
							Protection against type 2 diabetes from resistin, fucosidases
Ce	46	36	82	*SPG7*, *ERCC3*, *COL5A3*, *GSTM2*		High Axis 6	
Cf	19	41	60		*TPM1*, *KCNH2*, *SIAE*, *IRAK3*	High Axis 5	Negative regulation of immunity with high IL6, IL8
							High *TPM1*, *KCNH2* with hypertension
Cg	0	1	1				
Ch	28	65	93		*GSTP1*, *MTHFR*, *TNF*, *CEBPA*		
Mi	38	80	118	*ADSL*, *GNE*, *ARMD3*, *DISC1*	*CLN6*, *FTL*	Low Axis 6	*FTL* with high ferritin, total iron capacity
						High Axis 2	High Axis 2 with high red blood cell count
Mj	17	31	48	*F5*, *FCGR2C*, *FCGR3A*, *FCGR3B*, *LILRA4*	*CD8A*, *CCR5*, *IL2RB*, *IL12RB2*	High Axis 1	Unusual profile predicts aberrant immune signaling
Mk	3	9	12		*SNTA1*, *FADS2*, *COCH*		
Ml	14	12	26	*FANCA*, *SIDT2*, *SLC22A18*	*ENC1*	Low Axis 3	Four genes are biomarkers of leukemia in the observed direction

In several instances, also documented in Table 1, specific examples of aberrant gene expression are plausibly related to blood traits. For example, gene ontology analysis shows that Aa had up-regulation of 17 genes (*p* < 10^−5^, hypergeometric test) known to be responsive to the active form of vitamin D, calcitrol—a possible response to low blood calcium. Mi had high expression of the ferritin light chain gene, *FTL*, and correspondingly high serum ferritin and total iron capacity. Ab had impaired glucose tolerance and low expression of several blood glucose transporters; failed to express three genes in the homocystinuria type Cb1C pathway, which may explain her abnormally low glutathione and cysteine-glutathione levels; and showed up-regulation of *TUBB1*, consistent with high platelet counts because loss of function of the gene is reported to lead to thrombocytopenia. Ad had a particularly interesting profile that is discussed further in the “Discussion” section. A few instances where the gene expression did not correspond, superficially, to expected phenotypes were also observed. For example, Ch had abnormally high expression of the *TNF* gene but it was Ac who has elevated TNFα serum levels, while Mj had loss of expression of multiple genes related to platelet function, but normal platelet levels.

For seven of the nine individuals with more than 50 extreme expression values, differential expression along one or more of the Axes was likely to be a major contributor to the deviation. For this analysis, we required that the individual had the highest or lowest axis score in the sample, and that PC1 of their differentially expressed genes be highly correlated with that of the ten blood informative transcripts for the corresponding axis. Thus, Aa expression correlated with low Axis 1, and with a very low lymphocyte to neutrophil ratio. Cf had the opposite ratio and correspondingly high expression of Axis 5, and Mj had high expression of Axis 1 leading to gain of notable T cell signaling genes such as *CD8*, *CCR5*, *IL2RA*, and *IL12RB*. Mi had high Axis 2, which is enriched for erythropoiesis, and had high red blood cell counts, while Ml expressed four genes in directions expected of altered expression in leukemia (*ENC1* up-regulated; *FANCA*, *SIDT2,* and *SLC22A18* down-regulated). Other examples are listed in Table 1, and it is notable that even though the axes are defined by positive covariance of gene expression, the enrichments at either end of the distribution were in most cases reflecting high or low expression in the expected direction: in other words, genes that were negatively correlated with an axis could also be differentially expressed. This is also readily apparent in Fig. 4a, because some individuals exhibited reciprocal patterns of gain or loss of expression. However, importantly, association with an axis was not sufficient for extreme expression, as in very few cases was one of the blood informative transcripts that define each axis individually aberrant, and clearly hundreds of other genes in the relevant axes were also not significantly divergent by ANOVA. Thus the coordinated regulation of gene expression contributes to abnormality but other factors are required to push the expression to an extreme.

Methylation does not appear to be one of those factors. Because all peak CpG were significantly different among individuals at *p* < 10^−4^, we focused the analysis on sites that were more than 0.3 beta units in each individual from the average beta of the remaining individuals, yielding 2,113 CpG sites listed in Additional file 1: Table S9. Of these, 705 were divergent in two or more individuals, reflecting bimodality of methylation that was not observed transcriptionally; in a few cases, all 12 individuals were deviant from the whole sample mean. Of the remaining CpG that were deviant in just a single person, 1,181 were hypomethylated and 227 hypermethylated (Fig. 4b). This bias was particularly strong in the African American women as is apparent in Fig. 4b. However, there was no significant overlap in the identities of the extreme genes that were both divergently methylated and transcribed (7 % observed versus 11 % expected proportion of peak CpG genes that were individual-biased).

We also asked whether there may be overlap in the pathways represented by genes at the extremes of the transcript and methylation profiles for each individual. Additional file 1: Table S10 lists each of the pathways enriched by gene ontology analysis using the ConsensusPathDB server [35, 36] to query KEGG, Reactome, Wikipathway, and other gene sets. Considerably more pathway enrichment was observed for the transcript than methylation data. In a few cases the joint analysis provided evidence for additional enrichments, notably for individual Ac, in estrogen receptor mediated signaling, glucose transportation, and kidney function. Parallel queries of the String database of protein–protein interactions generated large interaction networks for most individuals, but these were no more connected than networks generated from the same number of random gene sets of an equivalent size.

**Fig. 5:**
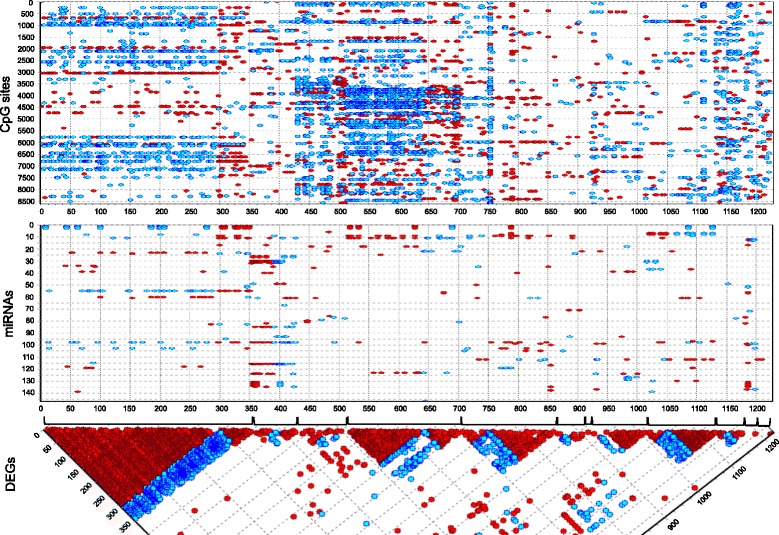
Correlation between differential gene expression, methylation, and miRNA abundance. *Red points* represent positive correlations, *blue* negative, at *p* < 10^−5^. The *lowest panel* shows the differentially expressed genes for each individual (ordered from left to right as Aa through Ml), nine of whom had a small number of genes negatively correlated with the major set of co-regulated transcripts that were extreme in that individual. The *middle panel* shows which of 150 miRNA correlated with the transcripts, including several species of miRNA that were either positively or negatively correlated with the extreme transcript sets in two or more individuals. The *top panel* shows methylation sites within 2 kb of the TSS that correlated with transcripts; these were almost all unique to one individual

There is some evidence that miRNA may be mediating some of the expression differences. Although miRNA are thought to down-regulate expression of genes by binding to the 3′ UTRs of target transcripts and destabilizing the message, cancer studies have demonstrated that the relationship between miRNA and mRNA abundance is complex and that there are as many positive as negative correlations [37–40]. That was also the case here: 42 miRNA correlated with aberrant gene expression profiles in multiple individuals, and another 37 in a single individual (Fig. 5). The figure indicates significantly positive and negative correlations based on the extreme gene expression measures ordered by individual along the x-axis. Wherever a methylation or miRNA was associated with one transcript, it tended to be associated with all of the extreme transcripts in that individual because they shared the same deviation. With the exception of Ad and Mj, there were three or fewer methylation-transcript abundance associations, and in all instances they were unique to one person. By contrast, six miRNAs were correlated with extreme transcripts in multiple individuals, even though they were only associated with a subset of the target genes in each individual. These miRNA–mRNA correlations are listed in Additional file 1: Table S11.

### Allele-specific individualized gene expression

In addition to overall transcript abundance, it is relevant to ask to what extent the ratio of transcription from the two chromosomes in each individual is maintained. ASE must be due in part to cis-acting influences on transcription, whether genetic or epigenetic, and has been shown to correspond broadly to eQTL effects. However, detailed cross-tissue comparisons imply that the regulation is also context-specific and often influenced by factors whose existence is inferred but not directly observed [32]. RNA-Seq data provide an opportunity to address the maintenance of ASE.

Sufficient read depth was available to survey ASE in nine of the participants (see “Methods” for description of cutoffs and procedures to account for alignment biases [41]; we report here alignments to each individual’s own genome as these are closer to the expected transcriptome-wide absence of bias). Whole genome sequences were previously generated [9], allowing independent identification of heterozygous sites in exons, an average of 6,971 of which were detected with at least eight reads in each of the three biological replicate RNA-Seq samples per individual. The standard deviation of the proportion of reads of the reference genotype was approximately 8 %, which implies that a single measure from one sample is an unreliable estimate of deviation from the expected 50:50 ratio of reference and alternate genotypes. However, the three samples did provide some power to evaluate deviations assuming binomial sampling and, using a two-tailed 5 % cutoff (|t| > 4.3, 2 degrees of freedom), 10.8 % of the heterozygous sites showed an allelic bias (range 9.4–12.7 %, with a trend for more sites with higher sampling depth, and approximately the same proportion observed with the reference genome alignment). One half of these significant biases had a deviation in favor of either allele of more than 10 %, and 20 % had a deviation of 20 % or more. The proportion of sites where ASE was observed both when the alignment was to the reference and to the alternate genome was just under 5 %, so formally not greater than expected by chance, but given the technical and sampling errors as well as restriction of the analysis to heterozygous sites, this was almost certainly an underestimate. Notably as well, up to 25 transcripts per individual were essentially monoallelic, with fewer than 5 % (usually none) of the reads from one of the alleles. Occasionally, one of the three samples was highly deviant, but it was not possible using these data to assess whether these cases represent biological effects or technical artifacts.

Pooling the heterozygous transcribed sites measured in at least three of the nine individuals resulted in 7,993 comparisons of the consistency of ASE bias. ANOVA was used to evaluate differences in the proportion of reference alleles among individuals, resulting in an estimated true negative rate, π_0_, of 81 %, and 325 significant differences at *p* < 0.01, a four-fold enrichment. The vast majority of these cases showed a range of biases among individuals, including one or more unbiased individuals (Additional file 1: Table S12). In addition, 16 cases where one individual was a clear outlier relative to the others were observed, as well as 40 cases where deviations from 50:50 were always observed to a similar magnitude but in both directions (consistent with loss of linkage disequillibrium between an eSNP and the transcribed SNP). For example, position 4534608 on Chromosome 17 had just 7 or 8 % alternate allele transcripts in Ab, Ff, and Fh, but 85 % in Mj (it was homozygous in the remaining individuals). These results, summarized in Additional file 1: Table S12A, suggest that while regulatory genotypes are a common source of ASE, their effects are not necessarily consistent among individuals, and hence other factors also modulate individual-specific ASE [42]. On the other hand, in the pairwise comparisons of the several thousand sites shared by any two individuals, the correlation of the magnitude of ASE effect was always highly significant and in the range of 0.4–0.5, further indicating that ASE effects are considerably more prevalent than those detected by our binomial sampling approach.

## Discussion

The data presented in this study support the notion that at the functional genomic level people have strongly persistent peripheral blood profiles. The temporal conservation is on a par with that observed for anthropometric and biochemical traits, so we refer to the profiles as a person’s “omic personality”, implying that we are each as molecularly individualized as we are morphologically and behaviorally. Despite considerable fluctuation in blood cell counts, technical measurement error, and well-documented influences of the environment [43], more than three quarters of the variation in the abundance of individual transcript was among people. Consequently, while not unique, our overall transcriptome profiles are recognizable from month to month, over the course of at least a year in healthy people.

This notion extends to the epigenome. Overall methylation profiles were almost as stable as transcriptional ones over the course of a year, though it is well documented that they change more over decades [11–18]. The proportion of variance in individual CpG methylation that was among individuals was lower than for transcripts, but most genes had at least one site that was more or less methylated in a subset of individuals, and collectively these generated an epigenomic personality. Intriguingly, there was little correspondence between the transcriptome and methylome in the sense that different pairs of individuals were more similar to one another for the two data types, and in the majority of cases where both transcript abundance and methylation of a single gene were strongly individualized, the two measures were not correlated. There were, however, several hundred loci where the correspondence was strong. Recent cross-sectional profiling across different classes of chromatin motifs support the inference that differential methylation often follows, and presumably stabilizes, activation of gene expression, though the relationship seems to be complex and context-dependent across tissues [35, 44–47]. Our results are consistent with this model, but emphasize that the relationship is restricted to a minority of genes, and that other mechanisms must be responsible for the long-term stability of most gene expression levels.

One such mechanism may be miRNA profiles. These were surprisingly less stable in this study than the other two omic measures, and it would not be possible to identify a person from their miRNA personality. This suggests miRNAs must work collectively to modulate gene expression; otherwise, the broader fluctuations in abundance would feed forward to the mRNA levels. A recent study [48] of mouse embryonic stem cells found that genes regulated by multiple miRNA tend to have lower noise at the protein expression level, but suggest that the effect is also a function of overall expression level, being stronger for lowly expressed genes. Several miRNAs did appear to correlate with the more extreme abundance levels of multiple individuals, consistent with a regulatory role, but one that involves a complex mix of positive and negative regulation as observed in many cancer genome anatomy studies. Mathematical modeling will be required to evaluate whether and how strongly miRNA can contribute to the temporal stability of mRNA and protein levels. There are surprisingly few studies of the global relationship between miRNA and RNA abundance in human population samples [26, 27], so we were unable to contrast our observations with an external dataset.

What are the consequences of omic personality for an individual’s health? Two aspects of personal transcriptomes are particularly noteworthy: the coordinate regulation along conserved axes of covariance, and the nature of expression at the extremes.

**Fig. 6:**
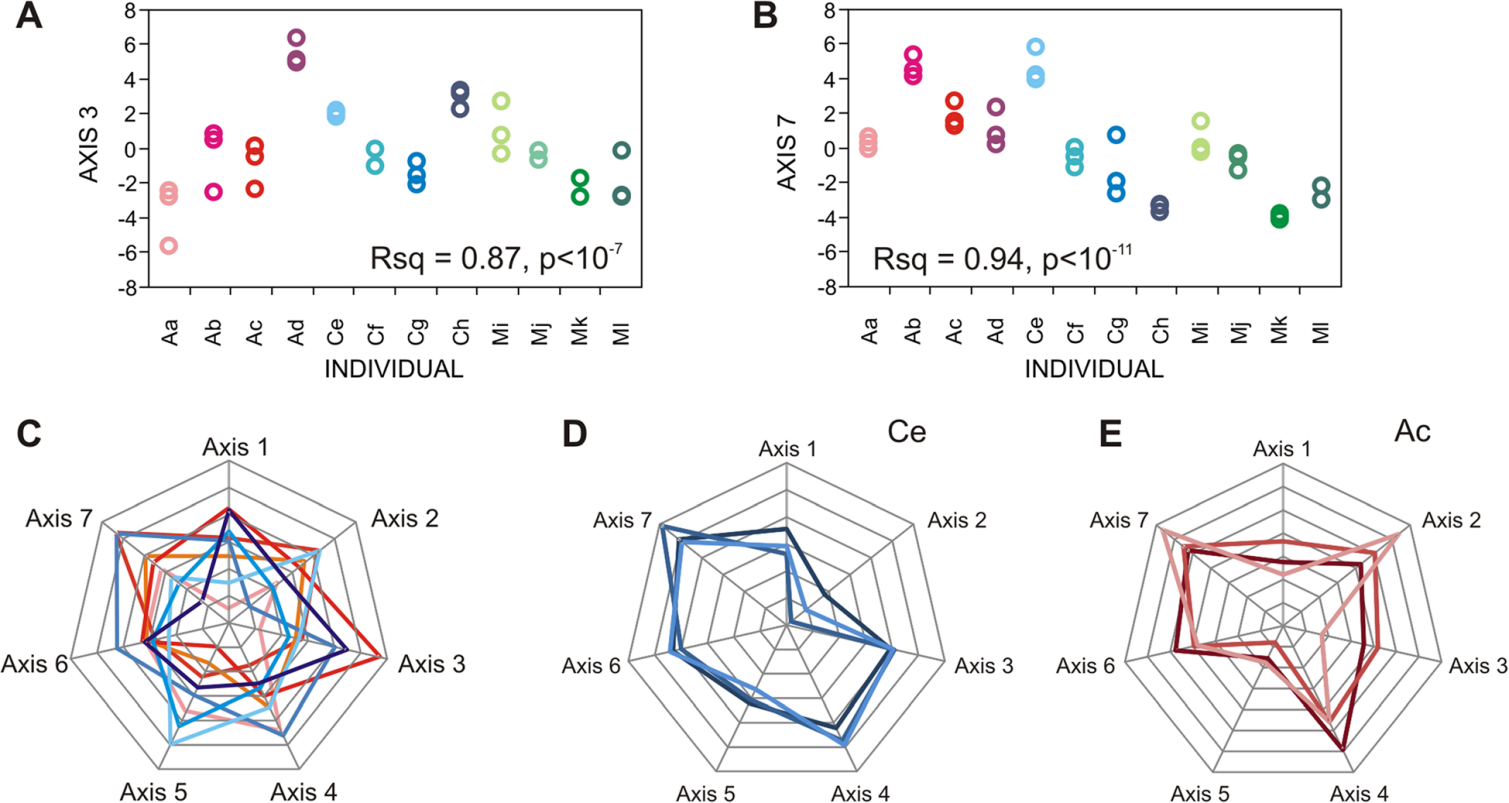
Variation in Axes of variation among individuals. *Top* PC1 scores for the ten blood informative transcripts corresponding to **a** Axis 3 or **b** Axis 7 for the three biological replicates of each individual, showing that 87 % and 94 % of the variance in the scores was among individuals. *Bottom* Radar plots showing standardized Axis score for the seven major Axes indicated with negative values near the center, high positive value the periphery. **c** Average score for each woman in the study. **d** Three consecutive score profiles for individual Ce, showing consistency over time. **e** Three consecutive score profiles for individual Ac, showing deviation for two axes at the final sample (*pink*), suggesting a deviation in her health at that time

We have recently noticed that in cross-sectional peripheral blood gene expression profiling datasets, between one half and two thirds of the variance in transcript abundance is explained by correlation with seven major axes, each defined by the covariance of ten blood informative transcripts [24]. The relationship held in this relatively small study of 12 people, such that each person had an individualized axis profile as shown in Fig. 6 based on data in Additional file 1: Table S13. The radar plots provide a summary peripheral blood profile for each person, which we believe can be used to as a baseline to evaluate a person’s overall immune status. Four of the axes are enriched for functions related to T-lymphocyte activity, B-lymphocyte activity, inflammation, and the interferon response (Axes 1, 3, 5, and 7 respectively), another (Axis 2) is consistently associated with obesity [49], as well as anxiety [50], while the roles of Axes 4 and 6 are yet to be clarified. As first observed by Chaussabel and colleagues [23, 51], covariance contained within these axes is perturbed in a variety of immune diseases, and similarly Cole and colleagues have defined a conserved transcriptional response to adversity that is also related to the axes and associates with multiple aspects of mental health [52, 53]. It has also been shown that baseline gene expression variation may predict vaccine response [54, 55], and we have identified a subset of Axis 1 that seems to be predictive of cardiovascular death in a cohort with extant coronary disease [56]. In unpublished work on the full CHDWB cohort, we observe many correlations of Axis scores with clinical measures. It is consequently hard to argue that the individualized profiles, reflecting regulation along gene expression axes, does not influence each person’s immune health to some degree—whether this is clinically useful remains to be clarified.

Eleven of the 12 participants described here displayed several dozen to several hundred individual gene expression measures that can be considered extreme, because they were more than two standard deviation units divergent from the expression levels observed in the other participants. Up to half of these instances of deviant expression can be explained in part by the covariance along one or more of the axes, some can be attributed to cis-eQTL and epigenetic factors, and it is likely that cumulative trans-eQTL effects are also contributing. In several cases, multiple genes with related immune or blood functions were indicated, and correspondingly the individual was the most extreme for the relevant trait, as documented in Table 1. A caveat to this analysis is that evaluation of any one individual was necessarily performed by comparison with a panel of others who may have been profiled at a different time, and that other experimental design issues could lead to technical confounding which may exacerbate differences. Nevertheless, results such as these suggest that individualized longitudinal gene expression profiling has considerable potential to contribute to personalized medicine assessments.

Assessments at *N* = 1 cannot, by definition, be supported by statistical evaluation. The observation of one or a handful of observations that make sense, picked from thousands of possible such evaluations, does not constitute a robust argument that the elevation or loss of a particular gene explains a phenomenon such as low blood pressure or elevation of a serum analyte. However, it is also the case that genetic risk scores based on rigorously established genome-wide association study evidence rarely have convincing positive predictive value, even where their sensitivity is high [57–59]. This is partly because those associations are based on population averages, and ultimately the effects of polymorphisms and mutations need to be assessed within the context of an individual. Our contention is that gene expression and, where possible, proteomic, metabolomic, immune, and other types of profile will add substantially to our understanding of why each person has disease susceptibilities, or,as importantly, is protected from disease [60, 61].

As an illustration of the latter, consider the profile of participant Ad. This individual was in her mid-60s and had a very high percentage of body fat with a body mass index in the overweight to obese range, yet her blood glucose and triglycerides were perfectly normal. A possible contributing factor was that she had aberrantly low expression of two genes involved in the breakdown of fucose to glucose. By contrast, her aberrantly high expression of the transcript encoding the myeloid-secreted adipocytokine resistin [62] was not prima facie consistent with her mid-range low-density lipoprotein cholesterol or the absence of any signs of diabetes [63]. This peptide is also pro inflammatory, but participant Ad actually had the lowest Axis 5 score in the study, consistent with generally low inflammatory activity including normal IL-6 and TNFα levels. Nevertheless, the transcriptome profiling might be clinically relevant if consideration were given to placing her on statins to treat her high blood pressure, given evidence that serum resistin opposes statin-mediated reduction of cholesterol production in the liver in overweight people [64]. A major challenge for integrative genomic profiling will be to be able to move beyond association of lists of genotypes or transcripts that associate with traits, to predictive models that explain how personal profiles relate to individuals’ unusual conditions. While not definitive, such models may be regarded as hypothetical causal relationships for further investigation, much as predicted deleterious rare alleles need additional validation before they can be assumed to be pathogenic.

An important aspect of this endeavor will be recognizing that functional genomic measures are characterized by strong patterns of co-regulation. This is in contrast to genotype-oriented studies that assume explicitly that genes are regulated independently of one another. This follows from the fact that disease-associated SNPs in different genes are overwhelmingly in linkage equilibrium with one another (disregarding population structure). That is patently not the case for transcripts. For reasons we dimly understand, the transcriptome, and presumably proteome, is designed to produce stable patterns of gene activity uniting hundreds and, in some cases, thousands of gene products. These patterns are sometimes correlated with phenotypes, including disease susceptibility, in cross-sectional studies, but their utility for personalized medicine will be a function of their stability over time. Methylation profiles seem to change more as people age than do transcriptome profiles, partially in response to changes in cell type abundance, so this must be accounted for, and they may be more difficult to interpret in the context of assessing changes in health status. On the other hand, once we accumulate more knowledge of how tightly modules and axes of genes are co-regulated, deviations of individual transcripts and methylation states may be assessed relative to those genes that remain stable. More extensive longitudinal profiling will indicate which genes have high coefficients of variation, and which are the most stable and hence most informative with respect to an individual’s health and disease.

## Conclusions

Although this study only considers healthy people over the course of a single year, it is clear that people express an “omic personality” consisting of peripheral blood transcriptional and epigenetic profiles that are relatively constant and reflect various types of immune activity. Some of these people are likely to go on to develop chronic conditions in the next 10 to 20 years. Ongoing profiling may allow us not just to ask how these profiles associate with clinical features, but also to address the major issue of whether baseline profiles predispose individuals to disease, or more commonly provide a level of homeostasis that protects them from perturbations that might otherwise push them into a state of ill health.

## Additional files

Additional file 1: Table S1. Amount of variation among individuals. **Table S2.** Experimental design file. **Table S3.** Principal components of the omic measures. **Table S4.** Methylation R-squared statistics by CpG location and type. **Table S5.** Correlations between peak CpG and nearest transcript abundance. **Table S6.** Correlations with cell counts. **Table S7.** Correlations with Axis scores. **Table S8.** Extreme transcripts by individual. **Table S9.** Extreme peak CpG by individual. **Table S10.** Enriched pathways in extremes of expression and methylation. **Table S11.** Correlations between miRNA and mRNA. **Table S12.** Allele-specific expression. **Table S13.** Axis scores (PC1 of the blood informative transcripts). (XLSX 6779 kb) Additional file 2: Figure S1. Comparison of allele-specific alignment biases to reference and alternate genomes. (PDF 891 kb)
