# Supplementary figures and images for: Omic personality: implications of stable transcript and methylation profiles for personalized medicine
Source: Genome Med. 2015 Aug 13;7(1):88. doi: 10.1186/s13073-015-0209-4 (PMC4578259; doi:10.1186/s13073-015-0209-4)

Figure S1. Tabassum et al, 2014

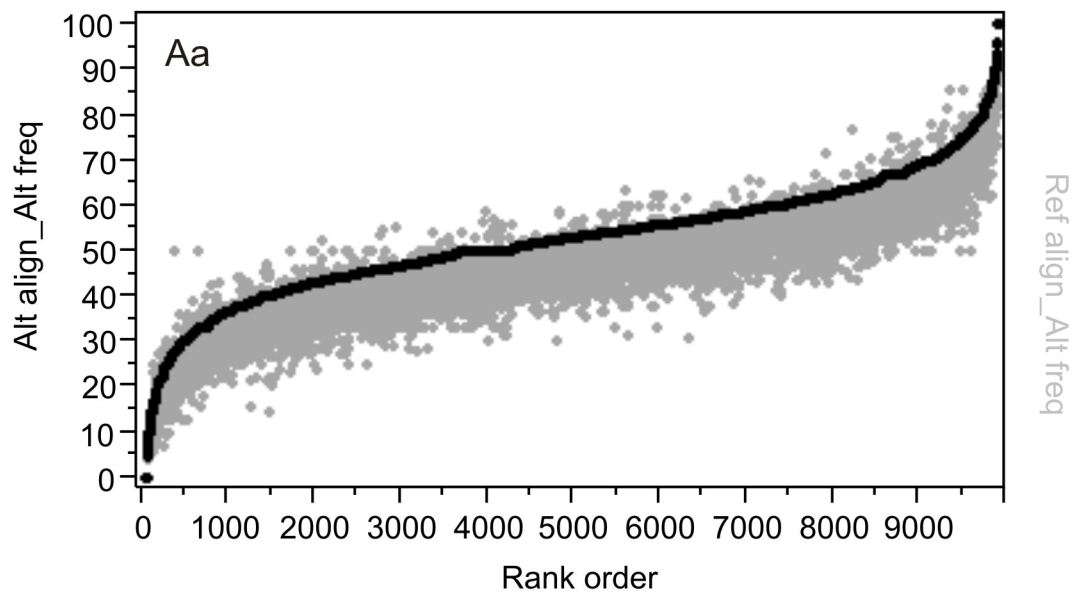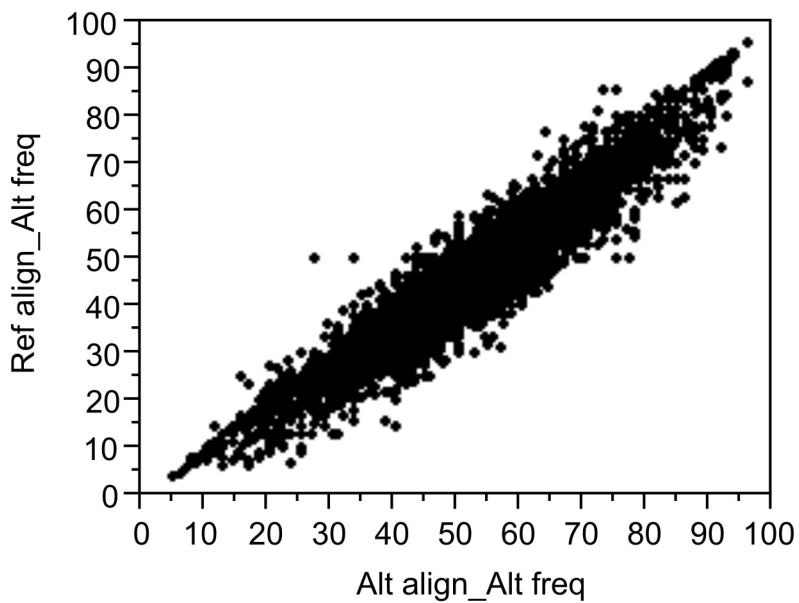

Supplement: Additional file 2: Figure S1. — Comparison of allele-specific alignment biases to reference and alternate genomes. (PDF 891 kb) [file 13073_2015_209_MOESM2_ESM.pdf]
